# Supplementary material for: Biopsychosocial Response to the COVID-19 Lockdown in People with Major Depressive Disorder and Multiple Sclerosis
Source: J Clin Med. 2022 Dec 1;11(23):7163. doi: 10.3390/jcm11237163 (PMC9738639; doi:10.3390/jcm11237163)
Supplement: Supplementary file 1 [file jcm-11-07163-s001.zip › jcm-1982303-supplementary.pdf]

Supplementary Materials

Table S1. Level of completeness of the data for the cohort of MDD and MS.

	MDD (N = 255)		MS (N = 213)	
	Valid N (%)	Observations N	Valid N (%)	Observations N
**PHQ-8**	255 (100)	691	213 (100)	479
**HR variables**	234 (91.76)	571	193 (90.61)	419
**Steps**	218 (85.49)	561	192 (90.14)	446
**Sedentary, activity light, moderate, and vigorous**	225 (88.23)	593	199 (93.43)	446
**Social contacts**	216 (84.70)	512	157 (73.71)	288
**Social interactions**	212 (83.14)	491	172 (80.75)	337

Note: Number of participants for each group who have data for the studied periods: pre-lockdown, during lockdown and post-lockdown. All variables of HR have the same N of Ids, as well activity (sedentary, light, moderate and vigorous intensity).

Table S2. Estimated mean differences in each outcome between no or mild depression vs moderate or severe depression at each period and interaction with gender in MDD group.

	**Pre lockdown Estim (95% CI, *p*-Value)**	**During-Lockdown Estimate (95% CI, *p*-Value)**	**Post-Lockdown Estim (95% CI, *p*-Value)**	**Pre-vs during Lockdown, Baseline Depression Severity and Gender (Interaction *p*-Value)**	**Pre vs Postlockdown, Baseline Depression SEVERITY and gender (Interaction *p*-Value)**
**PHQ-8 (N= 255)**					
**No or Mild Depres vs Mod-Severe Depres**	**−9.44 (−10.69 to −8.18) *****	**−9.38 (−10.84 to −7.93) *****	**−8.21 (−9.74 to −6.67)*****	**0.027**	0.197
**Steps (N=218)**					
**No or Mild Depres vs Mod-Severe Depres**	**1.16 (0.21 to 2.11)** *	1.06 (0.09 to 2.03) *	0.98 (−0.09 to 2.05)	0.570	0.936
**Sedentary(N=217)**					
**No or Mild Depres vs Mod-Severe Depres**	−34.02 (−139 to 71.0)	−46.34 (−174 to 80.9)	−4.47 (−133 to 123.6)	0.706	0.812
**Light Activity**					
**No or Mild Depres vs Mod-Severe Depres**	17.64 (−8.84 to 44.1)	7.78 (−18.02 to 33.6)	15.54 (−12.42 to 43.5)	0.847	0.772
**Moderate Activity**					
**No or Mild Depres vs Mod-Severe Depres**	1.95 (−3.47 to 7.37)	2.66 (−1.74 to 7.05)	1.12 (−3.80 to 6.03)	0.294	0.182
**Vigorous activity (N=217)**					
**No or Mild Depres vs Mod-Severe Depres**	**7.80 (2.69 to 12.9)** **	**5.33 (0.09 to 10.6)** *	**7.91 (2.31 to 13.5)** **	0.243	0.456
**mHR /day (N=234)**					
**No or Mild Depres vs Mod−Severe Depres**	−1.34 (−4.14 to 1.47)	−0.79 (−3.48 to 1.88)	−0.33 (−3.32 to 2.66)	0.378	0.222
**Std HR /day (N=234)**					
**No or Mild Depres vs Mod-Severe Depres**	0.67 (−0.34 to 1.68)	0.98 (−0.21 to 2.18)	**1.23 (0.08 to 2.39)** *	0.940	0.526
**Resting mHR day**					
**No or Mild Depres vs Mod-Severe Depres**	−2.11 (−4.92 to 0.69)	−1.34 (−4.01 to 1.32)	−0.90 (−3.90 2.08)	0.515	0.307
**Resting stdHR day**					
**No or Mild Depres vs Mod-Severe Depres**	0.01 (−0.83 to 0.85)	0.48 (−0.65 to 1.63)	**1.12 (−0.003 to 2.24)** *	0.789	0.896
**Resting mHR at night (N=234)**					
**No or Mild Depres vs Mod-Severe Depres**	−2.42 (−5.36 to 0.52)	−2.24 (−5.32 to 0.84)	−1.95 (−4.93 to 1.02)	0.096	**0.047**
**Resting stdHR night (N=234)**					
**No or Mild Depres vs Mod-Severe Depres**	0.177 (−0.44 to 0.79)	−0.09 (−0.77 to 0.57)	−0.28 (−1.03 to 0.45)	0.666	0.205
**Social contacts (N= 216)**					
**No or Mild Depres vs Mod-Severe Depres**	81.6 (−139 to 303)	82.6 (−141 to 306)	83.5 (−142 to 309)	0.226	0.224
**Social interactions (N=212)**					
**No or Mild Depres**	−1.28 (−4.07 to 1.50)	−1.57 (−5.93 to 2.79)	−0.61 (−4.23 to 3.00)	0.076	0.103

**vs Mod-Severe Depres**					

* $p < 0.05$; ** $p < 0.01$; *** $p < 0.001$ Note: **PHQ-8=** depression severity **Steps=** mean of steps per day, **Sedentary =** The number of minutes per day classified as a sedentary. **Light, moderate and vigourous activity**: the number of minutes per day classified as "lightly" or "moderate" and "vigorous "activity", **, mHR day**=mean HR during 24h, **std HR** = standard deviation of HR during 24h, **restingHR day**= HR at rest during 24h, **Resting HR at night**= HR at rest during the night (0:00-05:59), **social contacts** = number of contacts in the agenda, **social interactions =** interaction trough the social apps.

Table S3. Estimated mean differences in each outcome between no or mild depression (vs moderate or severe depression at each period and interaction with gender in MS group.

	**Pre Lockdown Estimate (95% CI, *p*-Value)**	**During-Lockdown Estimate (95% CI, *p*-Value)**	**Post-Lockdown Estimate (95% CI, *p*-Value)**	**Pre-vs during Lockdown, Baseline Depression Severity and Gender (Interaction *p*-Value)**	**Pre vs Post-Lockdown, Baseline Depression Severity and Gender (Interaction *p*-Value)**
**PHQ-8 (N= 213)**					
**No mild Depres Vs Mod-Severe Depres**	−7.00 (−8.29 to −5.72) ***	−7.03 (−8.42 to −5.65) ***	−7.59 (−8.96 to −6.23) ***	0.523	0.477
**Steps(N=192)**					
**No mild Depres Vs Mod-Severe Depres**	**1.15 (0.003 to 2.31)** *	0.83 (−0.34 to 2.01)	0.34 (−0.93 to 1.61)	0.322	0.127
**Sedentary(N=192)**					
**No mild Depres Vs Mod-Severe Depres**	15.4 (−133.6 to 103)	60.0 (−66.3 to 186)	−30.5 (−166.7 to 106)	0.241	0.712
**Light Activity**					
**No or Mild Depres vs Mod-Severe Depres**	31.08 (−9.72 to 71.9)	24.14 (−17.28 to 65.6)	7.39 (−38.89 to 53.7)	0.148	0.293
**Moderate Activity**					
**No mild Depres Vs Mod-Severe Depres**	3.49 (−4.26 to 11.2)	5.06 (−2.05 to 12.2)	4.35 (−2.65 to 11.4)	0.212	0.153
**Vigorous activity (N=217)**					
**No or Mild Depres vs Mod-Severe Depres**	5.30 (−1.42 to 12.03)	6.078 (−0.53 to 12. 69)	−0.15 (−7.55 to 7.25)	0.989	0.0827
**Social contacts (N= 157)**					
**No mild Depres Vs Mod-Severe Depres**	−22.7 (−174 to 128)	−32.2 (−186 to 122)	−41.3 (−203 to 120)	0.08	0.380
***Social interactions* (N=172)**					
**No mild Depres Vs Mod-Severe Depres**	−2.65 (−9.23 to 3.92)	−4.01 (−11.01 to 3.00)	−0.91 (−8.55 to 6.74)	0.810	0.233
**mHR /day (N=193)**					
**No mild Depres Vs Mod-Severe Depres**	0.54 (−2.31 to 3.39)	−0.22 (−3.32 to 2.88)	−0.28 (−3.35 to 2.78)	0.784	0.662
**Std HR day (N=193)**					
**No mild Depres Vs Mod-Severe Depres**	0.74 (−0.31 to 1.79)	0.78 (−0.51 to 2.09)	0.19 (−0.98 to 1.37)	0.814	0.443
**Resting mHR day**					
**No mild Depres Vs Mod-Severe Depres**	−0.007 (−2.92 to 2.90)	−0.19 (−3.30 to 2.93)	−0.52 (−3.63 to 2.58)	0.843	0.190
**Resting stdHR day**					

**No mild Depres Vs Mod-Severe Depres**	0.54 (−0.40 to 1.48)	0.77 (−0.43 to 1.98)	0.24 (−0.90 to 1.38)	0.743	0.903
**Resting mHR at night (N=192)**					
**No mild Depres Vs Mod-Severe Depres**	−0.26 (−3.42 to 2.89)	−0.36 (−3.87 to 3.15)	0.663 (−3.13 to 4.46)	0.595	0.161
**Resting stdHR night (N=192)**					
**No mild Depres Vs Mod-Severe Depres**	0.409 (−1.35 to 0.53)	−0.03 (−1.21 to 1.14)	−0.03 (−1.21 to 1.14)	0.587	0.616
**Social contacts (N= 157)**					
**No mild Depres Vs Mod-Severe Depres**	−22.7 (−174 to 128)	−32.2 (−186 to 122)	−41.3 (−203 to 120)	0.08	0.380
***Social interactions* (N=172)**					
**No mild Depres Vs Mod-Severe Depres**	−2.65 (−9.23 to 3.92)	−4.01 (−11.01 to 3.00)	−0.91 (−8.55 to 6.74)	0.810	0.233

* $p < 0.05$; ** $p < 0.01$; *** $p < 0.001$ **Note:** Note: **PHQ-8=** depression severity **Steps=** mean of steps per day, **Sedentary =** The number of minutes per day classified as a sedentary. **Light, moderate and vigourous activity**: the number of minutes per day classified as "lightly" or "moderate" and "vigorous "activity", **, mHR day**=mean HR during 24h, **std HR** = standard deviation of HR during 24h, **restingHR day**= HR at rest during 24h, **Resting HR at night**= HR at rest during the night (0:00-05:59), **social contacts** = number of contacts in the agenda, **social interactions =** interaction trough the social apps.
